# Influence of Sliding Time Window Size Selection Based on Heart Rate Variability Signal Analysis on Intelligent Monitoring of Noxious Stimulation under Anesthesia

**DOI:** 10.1155/2021/6675052

**Published:** 2021-06-05

**Authors:** Qiang Yin, Dai Shen, Qian Ding

**Affiliations:** ^1^Department of Mechanics, Tianjin University, Tianjin 300350, China; ^2^Department of Anesthesiology, Stomatological Hospital, Tianjin Medical University, Tianjin 300070, China

## Abstract

In recent decades, little progress of objective evaluation of pain and noxious stimulation has been achieved under anesthesia. Some researches based on medical signals have failed to provide a general understanding of this problem. This paper presents a feature extraction method for heart rate variability signals, aiming at further improving the evaluation of noxious stimulation. In the process of data processing, the empirical mode decomposition is used to decompose and recombine heart rate variability signals, and the sliding time window approach is used to extract the signal features of noxious stimulation, respectively. The influence of window size on feature extraction is studied by changing the window size. By comparing the results, the feature extraction in the process of data processing is valuable, and the selection of window size has a significant impact. With the increase of selected window sizes, we can get better detection results. But for the best choice of window size, to ensure the accuracy of the results and to make it easy to use, then, we need to get just a suitable window size.

## 1. Introduction

The clinical anesthesiologists must know whether the patient is sufficiently anesthetized to tolerate direct laryngoscopy and tracheal intubation. In the absence of an accurate and objective method to determine the level of general anesthesia, this can lead to conditions such as under- or overdose of anesthesia [1]. Patients may be adequately anesthetized for a given stimulus level, but when faced with other more intense stimuli, such as tracheal intubation, there is a risk of underanesthesia. Current anesthesia depth monitors are accurate in assessing the hypnotic component of general anesthesia but are less reliable in assessing the analgesic and reflex inhibition components of anesthesia [2]. We hope to use a more effective tool to solve this problem.

A significant relationship between the autonomic nervous system and many physiological factors modulating has been investigated during the last several decades. Heart rate variability (HRV) has been proposed as a method to be able to measure the stress response and the balance of analgesia and nociception in real-time when patients are under general anesthesia by assessing the autonomic nervous system [3, 4], making it possible to objectively evaluate noxious stimulation through HRV analysis. By comparison, HRV represents one of the most promising indicators. The easy derivation of this method has popularized its use. It is also seen as a simple tool for both theoretical research and clinical research. However, the significance of many different measures of HRV is more complex than generally appreciated and there is a potential for incorrect conclusions and excessive or unfounded extrapolations.

HRV analysis is a tool that can be used to observe the interaction of the sympathetic and parasympathetic nervous systems [5]. The quantification methods of HRV are categorized as follows: time domain, frequency domain, and nonlinear methods [6]. In time-domain analysis, the intervals between adjacent normal R waves are measured over the period of recording. Various statistical variables can be calculated directly from the differences between intervals and intervals. Traditionally, spectral analyses are always adopted in short-term studies, and often standard 5-minute electrocardiograph (ECG) segments are analyzed. A large number of nonlinear measures of HRV have been studied, but only a few have shown clear utility. Many practical applications of HRV analysis use different time domains, frequency domain, and nonlinear analysis techniques [7–10]. The features of HRV signals are of great and practical benefits for early diagnosis, monitoring, and prognostic assessments of the diseases [11, 12]. Combined efforts of clinicians and engineers have made it possible to use the data extracted from the heart rate variability to aid the diagnosis and prediction of various heart diseases as well as ailments originating from different human organs but indirectly influencing the autonomous nervous system. A lot of methods of nonlinear dynamics (e.g., estimating Lyapunov exponents) and complexity measures (e.g., correlation dimension or entropies) have been applied to HRV analysis [13–16]. In recent years, empirical modal decomposition (EMD) of nonlinear and nonstationary time series has been proposed. This signal analysis technique is used for the analysis of HRV signals and other signals [7, 8, 17–20]. To accurately extract the features of the signals, the time window method can be used to accurately set the size of the time window for more effective feature recognition [21]. In recent years, neural networks have received increasing attention for medical applications and are gradually being used in clinical practice, but direct application of neural networks has not led to more general conclusions in medical signal analysis, so aiding appropriate signal feature extraction may be more effective than specific classification methods [15, 22, 23]. Applying these research methods properly will give a great advancement to the research of revealing the underlying law and physical nature of HRV.

Based on the abovementioned physiological basis and research methods, the sliding time window method is introduced to extract the features of noxious stimulation in HRV analysis, thus aiding the classification method, and we need to study the effect of window size on feature extraction.

## 2. Materials and Methods

### 2.1. Data Collection

This study has been approved by the Ethics Committee of the hospital, and all patients have signed the informed consent. Sixty individuals (ASA grade I or II, all genders, age 18~60, BMI<30 kg/m^2^) have undergone oral and maxillofacial surgery under general anesthesia. Exclusion criteria: the diseases are known to affect autonomic nerve function.

ECG signals are continuously recorded by BMD101 (NeuroSky Inc.) at a sampling rate of 512 Hz and stored in the computer during perioperation. Signal processing is conducted to form the RR intervals to be analyzed. Three kinds of signals are obtained from the RR intervals: preoperative (T0), intubation (T1), and intraoperative (T2). In this study, all we need are the T1 and T2 signals. The T1 signals represent the occurrence of a noxious stimulation during anesthesia. T2 signals mean that only during anesthesia. The original RR intervals from the ECG signal are a function of the number of heartbeats instead of the time. To make the RR intervals be the function of the time, the signals are resampled using cubic spline at a sampling rate of 8 Hz as recommended for HRV studies [24].

### 2.2. Empirical Mode Decomposition

The EMD method is that any complicated signal can be decomposed into a finite and often small number of intrinsic mode functions (IMF). Since the decomposition is based on the intrinsic timescale of the signal, it is suitable for nonstationary signals [25]. The IMFs are computed by the sifting process, which is an iteratively detrending operation. To extract IMFs from a given signal *s*(*t*), the procedure is described as follows:

*Step 1:* confirm all the local extrema

*Step 2:* generated the upper envelope *s*_up_(*t*) and lower envelope *s*_low_(*t*) by the local maxima and minima by using the cubic spline

*Step 3:* calculate the means of the upper envelope and the lower envelope as *m*(*t*)

(1) $$\begin{array}{c} m\left(t\right)=\frac{s_{\text{up}}\left(t\right)+s_{\text{low}}\left(t\right)}{2}. \end{array}$$

*Step 4:* compute the IMF candidate by the difference between *s*(*t*) and *m*(*t*); it can only be considered as an IMF if it meets the sifting stopping criteria

(2) $$\begin{array}{c} s\left(t\right)-m\left(t\right)=h_{1}\left(t\right), \end{array}$$

(3) $$\begin{array}{c} h_{1n}\left(t\right)=h_{1\left(n-1\right)}\left(t\right)-m_{1\left(n-1\right)}\left(t\right), \end{array}$$

(4) $$\begin{array}{c} h_{1n}\left(t\right)=IMF_{1}\left(t\right). \end{array}$$

*Step 5:* compute the residue *r*(*t*) by the difference between *s*(*t*) and the IMF

(5) $$\begin{array}{c} r_{1}\left(t\right)=s\left(t\right)-IMF_{1}\left(t\right). \end{array}$$

*Step 6:* repeat the above steps until *s*(*t*) is decomposed into a finite number of IMFs and one residue, and the residue is either a steady trend or a constant.

(6) $$\begin{array}{c} s\left(t\right)=\overset{k}{\underset{i=1}{\sum }}IMF_{i}\left(t\right)+r_{k}\left(t\right). \end{array}$$

EMD decomposes the nonstationary signal into a finite set of IMFs without information loss or distortion [19]. In this study, IMFs need to be recombined according to requirements to become the feature signals.

### 2.3. Sliding Time Window Method

The sliding time window (STW) method is a very effective feature extraction method. However, determining the optimal value for the window size is an important and difficult problem. The size of the sliding time window will affect the effect of feature extraction. If the size is too large, features may be confused together, affecting the accuracy of the results and increasing the computational load. On the contrary, if the size is too small, the features cannot be extracted completely, so that better results cannot be obtained [21].

The appropriate size of STW can be selected according to the characteristics of the research object. The size range of STW is generally determined according to the target object or application requirements under the guidance of prior knowledge. For this study, we can further compare the effects of different sizes of STW on feature extraction, to establish the selection criteria. This is also possible to explore the relationship between window size selection and the autonomic nervous system.

### 2.4. Model Building

In this work, we are interested in how to accurately detect noxious stimulation during anesthesia. Firstly, RR interval signals are decomposed and recombined into high-frequency (HF) component signals and low-frequency (LF) component signals, corresponding to sympathetic and parasympathetic activity in the autonomic nervous system, respectively. Secondly, the signal features of noxious stimulation are reflected in LF component signals, and LF signals are processed by the sliding time window method for feature extraction. Finally, the feature is extracted as the input to the deep neural network to determine whether the noxious stimulation (tracheal intubation stimulation) occurs under general anesthesia.

The long short-term memory (LSTM) network is used in the deep neural network model. The deep neural network model has the capability of feature extraction, so the RR intervals are directly used as the input to the deep neural network, and the results can be obtained directly by training the deep learning model. The results obtained by using raw data directly are compared with those obtained by extracting features with different time window sizes. This comparison can reflect whether the features obtained in the data processing are representative and whether they have a significant impact on the results.

## 3. Results

During the study period, the RR interval signals are from 60 patients, and a total of 104 signals are selected for the investigation after the screening, which includes 42 T1 signals and 62 T2 signals. These signals are randomly divided into 71 for the training set, 15 for the validation set, and 18 for the test set.

**Figure 1:**
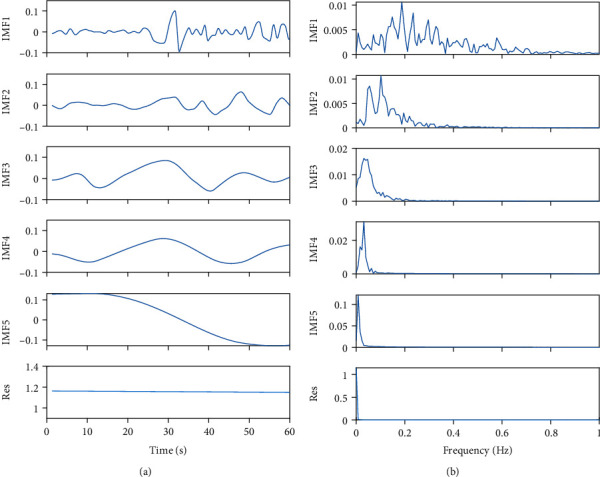
(a) The IMF components of the RR intervals. (b) Spectrum analysis on each IMF.

**Figure 2:**
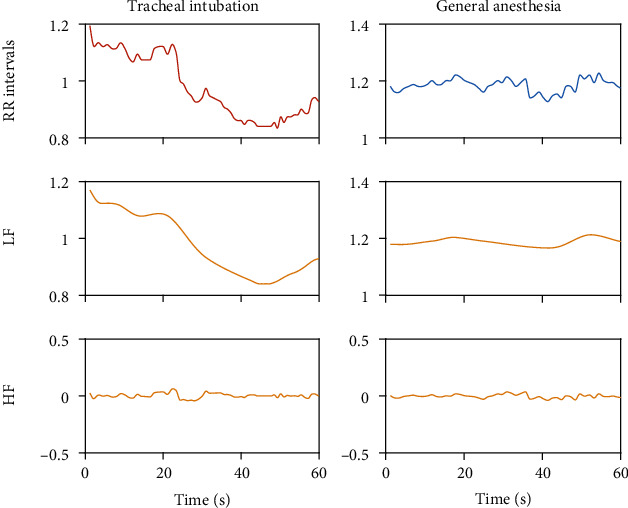
The original RR intervals, the HF component, and the LF component separately form two feature signals corresponding to autonomic nervous system regulation.

In the process of preprocessing RR interval signals, uniform time-domain sampling is obtained through cubic spline piecewise interpolation, which makes the RR series more suitable for feature extraction. In the frequency domain, the spectrum of the short-term HRV signal can be distinguished into several frequency bands [26]. These bands are referred to as the high frequency (HF) band (0.15 Hz to 0.4 Hz), the low frequency (LF) band (0.04 Hz to 0.15 Hz), and the very low-frequency band (VLF), i.e., bands less than 0.04 Hz [18]. Here, we combine the LF and VLF bands and consider them both as LF bands uniformly. Hence, using the EMD to decompose the resampled RR interval sequence into limited IMF components and performing spectrum analysis on each IMF, the results shown in Figure 1 indicate that the HF components (0.15~0.4 Hz) and LF components (0.04~0.15 Hz) are distinguished. By recombining the HF IMF component and the LF IMF component separately, two feature signals corresponding to autonomic nervous system regulation are formed, as shown in Figure 2. The HF band reflects only parasympathetic changes, while the LF band reflects changes in sympathetic and some other stress responses. Under general anesthesia, the HF component with tracheal intubation is similar to the HF component without tracheal intubation, and the LF components are significantly different.

**Figure 3:**
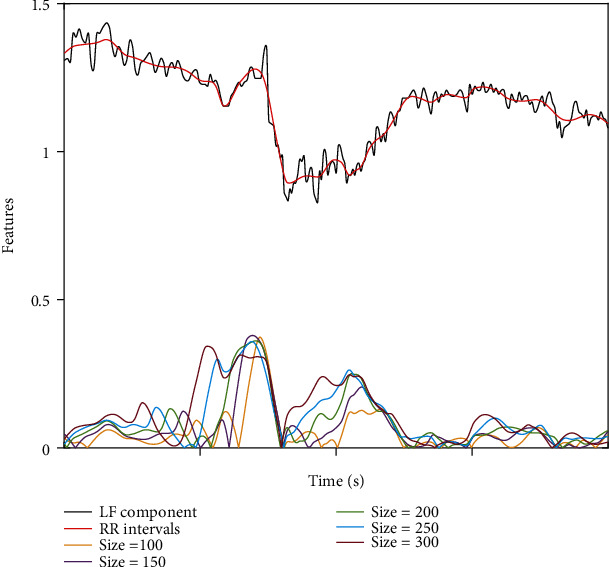
The extracted features corresponding to window sizes between 100 and 300 sampling points.

**Figure 4:**
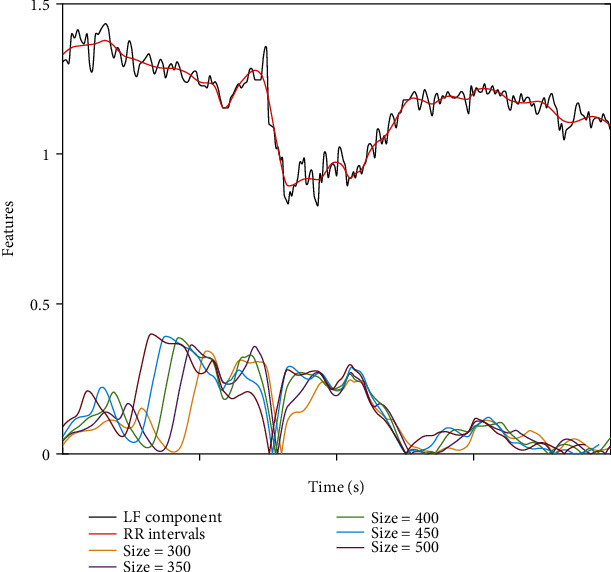
The extracted features corresponding to window sizes between 300 and 500 sampling points.

The LF component signals with the tracheal intubation stimulation have the obvious feature, and the feature extraction of noxious stimulation is completed within the STW, as shown in Figures 3 and 4. The extracted feature is the difference between the left endpoint and the right endpoint of the LF component signal within the STW. We can get different feature results for different window sizes, and by comparing these results, we can choose the best one. The minimum window size is 100 resampling points, and each increase of 50 sampling points is used to extract the feature results of the LF signal once. As shown in Figure 3, the window size is between 100 and 300 sampling points. We can find that the features of noxious stimulation have been extracted and the features become better and better as the window size increases. The extracted features have a corresponding change in amplitude when the noxious stimulation occurs. In Figure 4, we can see that as the number of sampling points increases gradually, the features that can be extracted become more obvious. This indicates that the window size directly affects the effect of feature extraction, and the larger the window size is, the more significant the effect is. For such a result, we finally select the features extracted from 150,250,350,450,550,650 sampling points to verify the effect.

**Figure 5:**
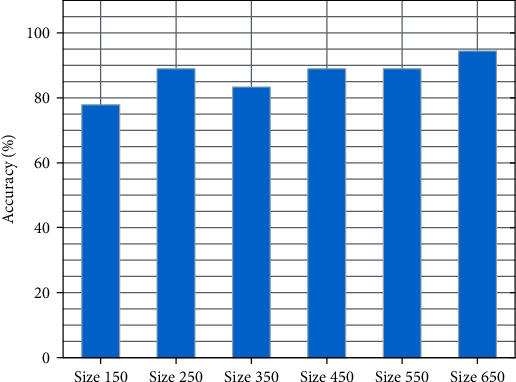
The accuracy of detecting the noxious stimulation during anesthesia with different window sizes.

Because the total number of signals we have is very limited, we have to verify the results multiple times for each window size. As shown in Figure 5, we can obtain that the accuracy of detecting the noxious stimulation corresponding to different window sizes is 77.8%, 88.9%, 83.3%, 88.9%, 88.9%, and 94.4%. By comparing the results, we conclude that the feature extraction in the process of data processing is valuable, and the selection of window size has a significant impact. If the window size is relatively small, the extracted features are concentrated in a small area of the signal, which makes the features of a small area of the signal less easy to capture. However, as the window size gradually increases, the STW method can easily capture the feature differences between different states.

**Figure 6:**
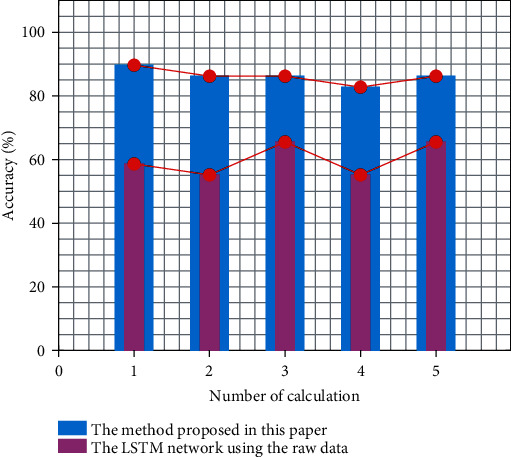
The accuracy of the LSTM network and the method proposed in this paper for training on the same dataset.

As shown in Figure 6, the data are directly fed into the LSTM network [27] for training on the same dataset, so that the corresponding results can be obtained. Due to the limited number of signals, the data set is randomly divided into training and test sets several times to obtain multiple results. Comparing with the results obtained by the method proposed in this paper, the results obtained by the method in this paper are more satisfactory.

## 4. Discussion

**Figure 7:**
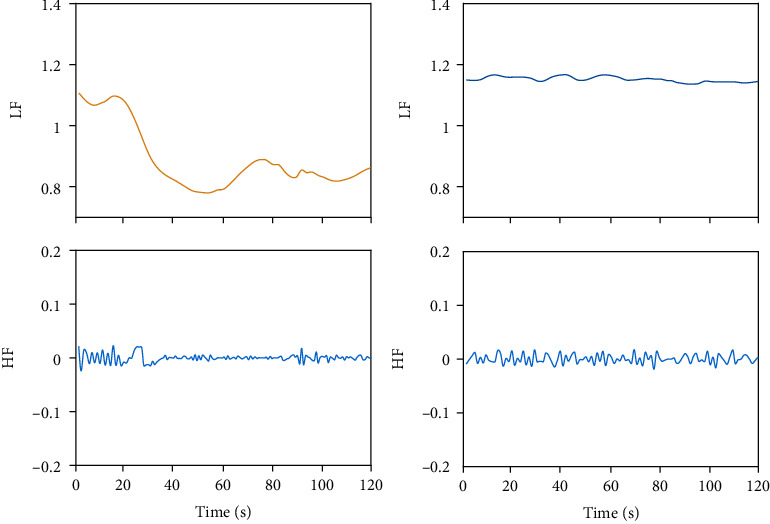
The feature of the signal: under general anesthesia, tracheal intubation stimulation will have an impact on the LF component.

In this study, ECG signals are collected under general anesthesia, and then, RR interval signals are obtained. HRV analysis includes time-domain, frequency-domain, and nonlinear analysis. Although these methods have been widely used, they still have limitations. HRV analysis has a good physiological basis for the evaluation of the autonomic nervous system; however, because HRV signals contain many complex components, no significant progress has been made in analytical methods and applications for many years. To solve this problem, the RR intervals are decomposed into the HF component and the LF component, corresponding to the autonomic nervous system. Under anesthesia, the HF component with the tracheal intubation stimulation is similar to the hf component without the tracheal intubation stimulation, and the LF components are significantly different, as shown in Figure 7.

In the research, the size of samples is relatively limited. To better address this limitation, it is very important to extract the common features of the signals. The quality of the signal features is also critical. If good enough signal features can be extracted, excellent results can also be obtained in the case of a limited number of signals. The conclusion of small sample data depends on the extraction effect of features.

Different sizes of STW have a definite effect on feature extraction. With the increase of selected window sizes, we can get better detection results. Although better results can be obtained when the window size is larger, it affects the real-time performance and practicability of detection. We should make the optimal choice under these conditions, it not only ensures the accuracy of the result but also makes it easy to use and operate, so we need to select the size of the window not to be too big. We also have to make sure that the effect is good and that the window size is chosen following autonomic nervous system regulation.

Although the method we proposed has achieved good results on this data set, the method of feature extraction is relatively single, which makes this method only applies to the situation of significant signal fluctuations, and more diverse feature extraction methods will be added in the future. Secondly, although the selection law of window size has been clear, different STW sizes may be adopted for signals in different states. Therefore, we need to further explore the adaptive STW to deal with signal feature extraction in various states. Finally, we will collect more signals for further research.

## 5. Conclusions

By comparing the results, we can find that feature extraction is valuable in data processing, and the selection of window size has a great impact. To balance the practical effect, it is necessary to choose an appropriate window size and follow the regulation rules of the autonomic nervous system.

## Data Availability

The data that support the findings of this study are available from the corresponding author on reasonable request.
